# Trends in type 2 diabetes mellitus disease burden in European Union countries between 1990 and 2019

**DOI:** 10.1038/s41598-021-94807-z

**Published:** 2021-07-28

**Authors:** Richard Goodall, Andrew Alazawi, Will Hughes, Vassiliki Bravis, Justin D. Salciccioli, Dominic C. Marshall, Conor Crowley, Joseph Shalhoub

**Affiliations:** 1 Imperial College London, London, UK; 2 Oxford University Hospitals NHS Trust, Oxford, UK; 3 Medical Data Research Collaborative, London, UK; 4 Barts and The London School of Medicine and Dentistry, Queen Mary University of London, London, UK; 5 Cambridge University Hospitals NHS Trust, Cambridge, UK; 6 Imperial College Healthcare NHS Trust, Imperial College London, London, UK; 7 Brigham and Women’s Hospital, Harvard Medical School, Harvard, UK; 8 Lahey Hospital and Medical Center, Burlington, USA

**Keywords:** Diabetes complications, Type 2 diabetes

## Abstract

This observational study aimed to assess trends in type 2 diabetes mellitus (T2DM) disease burden in European Union countries for the years 1990–2019. Sex specific T2DM age-standardised prevalence (ASPRs), mortality (ASMRs) and disability-adjusted life-year rates (DALYs) per 100,000 population were extracted from the Global Burden of Disease (GBD) Study online results tool for each EU country (inclusive of the United Kingdom), for the years 1990–2019. Trends were analysed using Joinpoint regression analysis. Between 1990 and 2019, increases in T2DM ASPRs were observed for all EU countries. The highest relative increases in ASPRs were observed in Luxembourg (males + 269.1%, females + 219.2%), Ireland (males + 191.9%, females + 165.7%) and the UK (males + 128.6%, females + 114.6%). Mortality trends were less uniform across EU countries, however a general trend towards reducing T2DM mortality was observed, with ASMRs decreasing over the 30-year period studied in 16/28 countries for males and in 24/28 countries for females. The UK observed the highest relative decrease in ASMRs for males (− 46.9%). For females, the largest relative decrease in ASMRs was in Cyprus (− 67.6%). DALYs increased in 25/28 countries for males and in 17/28 countries for females between 1990 and 2019. DALYs were higher in males than females in all EU countries in 2019. T2DM prevalence rates have increased across EU countries over the last 30 years. Mortality from T2DM has generally decreased in EU countries, however trends were more variable than those observed for prevalence. Primary prevention strategies should continue to be a focus for preventing T2DM in at risk groups in EU countries.

## Introduction

In 2019, the global estimate for the number of adults over 18 years of age with diabetes mellitus was 451 million^1^, with an estimated 5 million worldwide deaths attributable to diabetes among people aged 20–99 years^1^. Treating diabetes and its complications cost the National Health Service (NHS) in the United Kingdom (UK) an estimated £14 billion in 2018^2^, representing 10% of the annual NHS budget for England and Wales. Causal associations exist between obesity, unhealthy diet, physical inactivity and type 2 diabetes mellitus (T2DM)^1^. Comparisons of up to date prevalence and mortality outcomes from T2DM across European countries are lacking.

The principal aim of this study was to compare the trends in prevalence and mortality from T2DM in the countries of the European Union (EU) (inclusive of the United Kingdom). The study aimed to further assess the differential burden of disease across EU countries using disability-adjusted life-years (DALYs). We carried out an observational analysis of the Global Burden of Disease (GBD) database^3,4^ to assess prevalence, mortality and disability-adjusted life-years from T2DM in the EU countries between 1990 and 2019.

## Online methods

### Characteristics of the data source

The data for this observational analysis of T2DM was obtained from the GBD database, which amalgamates datasets from 127 countries to provide mortality and disability data (deaths, death rates, years of life lost due to premature mortality, prevalence and incidence) for a collection of the world’s most important health concerns, as commissioned by the World Health Organisation (WHO). The exact GBD methodology has been published previously^3,4^, and we have previously described this methodology in observational analyses of trends in the disease burden of peripheral arterial disease^5^, abdominal aortic aneurysm^6^, and lower extremity amputation^7^.

The GBD Study defines T2DM as a fasting plasma glucose (FPG) ≥ 7 mmol/L (126 mg/dL), or HbA1c > 48 mmol/mol (6.5%), or those currently treated with anti-hyperglycaemic drugs or insulin^3,4^. The GBD uses different data sources and models to estimate mortality, prevalence and DALY rates, for which detailed descriptions are available^3,4^.

Briefly, for estimates of disease mortality, the GBD Study assigns each death to its single underlying cause, i.e. the cause that initiated the series of events that lead to death. For the present analysis, T2DM-related deaths in the total population, listed as the underlying cause of death, is defined as International Classification of Diseases-Tenth Edition (ICD-10) code E11. ICD-10 codes for T1DM were excluded. GBD mortality data is registered into a causes of death database, combining mortality estimates derived from multiple different types of data source (including vital registration (VR), verbal autopsy (VA), cancer registry, police records, sibling history, surveillance, and survey/census). VR data obtained from the WHO mortality database represent the source for most cause-of-death data, and individual countries submit a compilation of data to the WHO each year. Further VR data are identified and obtained from country-specific mortality databases operated by official offices. Unspecified diabetes deaths are redistributed on the basis of regression, in which the true proportions of type 1 and type 2 deaths by age-sex-location-year are a function of the proportion of unspecified deaths, age, the age-standardised prevalence of obesity and an interaction term for age and obesity prevalence.

A further component of the GBD methodology for cause of death estimates includes identification and redistribution of deaths assigned to ICD codes that either: cannot be the underlying cause of death (e.g. senility or frailty); are an intermediate cause of death rather than the underlying cause (e.g. sepsis and heart failure); or lack specificity in coding (e.g. diabetes (type not specified) or unspecified cardiovascular disease). These are described by the GBD authors as garbage codes, and are re-distributed to a single underlying cause of death using a garbage code redistribution algorithm described by Naghavi et al.^8^.

To characterise the quality of the mortality data available in each country, the GBD authors grade the reliability of annual cause of death estimates on a 5-star scale. For the countries analysed in the present analysis, with the exceptions of Cyprus and Slovakia (2-stars and 3-stars, respectively), 15 EU countries scored 4-stars (Belgium, Bulgaria, Croatia, Czech Republic, Denmark, France, Germany, Greece, Luxembourg, Netherlands, Poland, Portugal, Romania, Slovenia and Spain), representing greater than 65% completeness of mortality data. The UK and the 10 remaining EU countries have 5-star data, demonstrating greater than 85% completeness of the data (Austria, Estonia, Finland, Hungary, Ireland, Italy, Latvia, Lithuania, Malta and Sweden).

For estimations of disease prevalence, the GBD uses systematic reviews, survey data, disease registries, hospital administrative data, claims, inpatient and outpatient data, and case notifications as data sources. Using these data as input, population prevalence estimates (with corresponding confidence intervals) are computed using the DisMod-MR 2.1 meta-regression tool. Multiple cross-walks were performed on the available data-sets prior to the analysis to establish a standard reference definition; fasting plasma glucose > 126 mg/dL/7 mmol/L, or HbA1c > 48 mmol/mol (6.5%), or being on relevant treatment.

DALYs are calculated by the GBD group as the sum of years of life lost (YLL) and years lived with disability (YLD). GBD methodology computes YLL by multiplying the standard life-expectancy at the age of death by the estimated number of deaths (per cause)^9^. YLD were calculated by multiplying the prevalence of T2DM by the disability weighting for T2DM (gathered from the GBD 2013 European disability weights measurement study)^13^. YLDs are then corrected for co-morbidities via a microsimulation process, before combining with YLLs to give estimated DALYs and their corresponding uncertainty estimates.

The produced information is available to the public and can be extracted via the GBD Results Tool (http://ghdx.healthdata.org/gbd-results-tool). We used this tool to extract age-standardised prevalence, mortality and DALY rates for diabetes mellitus for EU countries and the UK between 1990 and 2019.

### Handling of the GBD data

Age-standardised prevalence rates (ASPRs), age-standardised mortality rates (ASMRs) and DALYs per sex per 100,000 population for T2DM were extracted from the GBD Results Tool for each of the years between 1990 and 2019, inclusive, for the UK and EU countries. Extracting age-standardised rates improves inter-country comparability, because differences in the age-structure of different populations are accounted for. For all age-standardised rates, the GBD Study computes a standard population using a non-weighted average across a percentage of the population of all countries in each five-year age bracket (years 2010–2035) from the United Nations Population Division’s World Population Prospects (2012 revision)^9,10^. Absolute and relative changes in ASPRs, ASMRs and DALYs over the observation period (i.e. differences between the rates in 1990 and 2019) were calculated for each sex in each country.

### Statistical analysis

Joinpoint regression analysis was used to assess trends in the disease burden of T2DM. The Joinpoint software (Joinpoint Command Line Version 4.5.0.1) was provided by the United States National Cancer Institute Surveillance Research Program^11^. This software tracks trends in data over time (for the present analysis, ASPRs, ASMRs and DALYs), then fits the simplest model possible to the data by connecting several different line segments on a logarithmic scale. These segments are known as ‘Joinpoints’, with the simplest model (i.e. 0 Joinpoints) being a straight line. As more Joinpoints are added, each is tested for significance using a Monte Carlo permutation method. The software also gives estimated annual percent changes (EAPC) for each line segment (with corresponding 95% confidence intervals). Each EAPC is tested to establish if a difference from the null hypothesis of no change exists. Consequently, the final model consists of multiple Joinpoints, each representing a statistically significant (*p* value < 0.05) change in trend (increase or decrease), with each trend described by the EAPC and the associated 95% confidence intervals. The EAPC allows assessment of trend changes at a constant percent per year.

## Results

Over the 30-year period studied, changes were observed in T2DM mortality, prevalence and DALYs across the 28 EU countries.

### 2019 T2DM mortality

**Figure 1:**
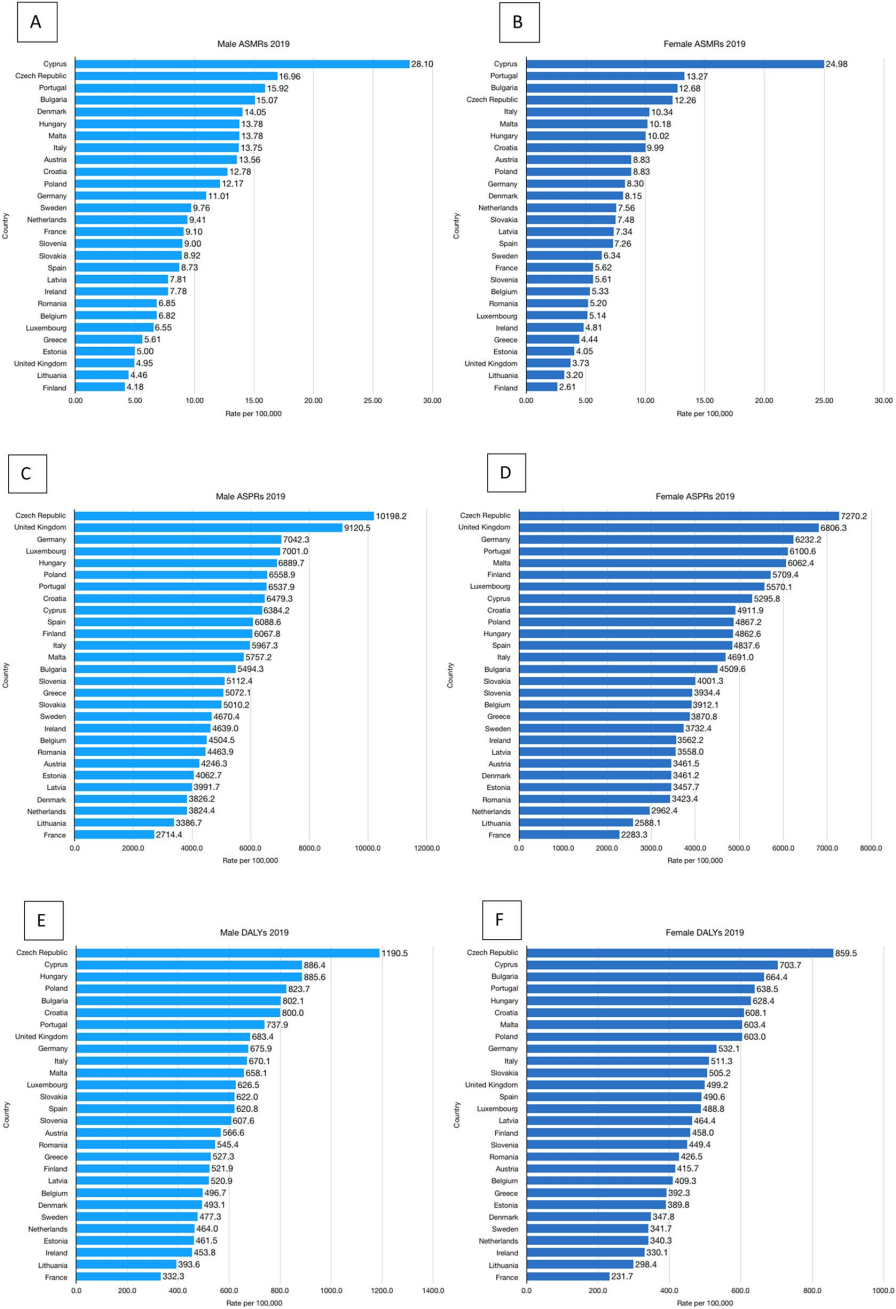
2019 T2DM disease burden. 2019 age-standardised mortality rates (ASMRs) (A&B), age-standardised prevalence rates (ASPRs) (C&D) and disability-adjusted life years (DALYs) (E&F) per 100,000 population for type 2 diabetes mellitus (T2DM) in all EU countries and the UK.

Data for the 2019 T2DM ASMRs per 100,000 for males and females in the 28 EU countries are demonstrated in Fig. 1. In 2019, the highest ASMRs were observed in Cyprus for both males and females (28.1 and 25.0/100,000, respectively). The lowest ASMRs were observed in Finland for both males and females (4.2 and 2.6/100,000, respectively). The 2019 ASMRs were higher in males than females in all 28 included countries. In the UK in 2019, the ASMR was 5.0/100,000 for males and 3.7/100,000 for females, which represented the third lowest ASMRs (for both sexes) of all included countries.

### 2019 T2DM prevalence

Figure 1 demonstrates ASPRs per 100,000 per country in 2019 for males and females, respectively. The highest ASPRs in 2019 were observed in the Czech Republic and United Kingdom for males (10,198.2 and 9,120.5/100,000, respectively) and females (7270.2 and 6806.3/100,000, respectively). France had the lowest ASPR in 2019 for males (2714.4/100,000) and females (2283.3/100,000). The 2019 ASPR was higher in males than females in all 28 countries.

### 2019 T2DM disability-adjusted life-years

Figure 1 shows the 2019 DALYs per 100,000 population for all 28 countries studied. The highest 2019 DALYs for both sexes were observed in the Czech Republic (1190.5/100,000 for males, 859.5/100,000 for females). The lowest 2019 DALYs were observed in both sexes in France (332.3/100,000 for males, 231.7/100,000 for females). In 2019, Male DALYs were higher than female DALYs in all 28 countries.

### Trends in T2DM mortality

Male ASMRs decreased in 16 of the 28 countries over the 30-year study period. For females, ASMRs decreased in 24 countries. The largest relative increases in ASMR between 1990 and 2019 in males and females were observed in Latvia (+ 82.5% for males, + 55.1% for females). The largest relative decrease in ASMR for males from 1990 to 2019 was in the UK (-46.9%). In females, the largest relative decreases in ASMR was identified in Cyprus (−67.6%). Females in the UK observed a −47.1% reduction in ASMRs over the period. This data can be found in Supplementary Tables 1a and 1b.

### Trends in T2DM prevalence

The overall trend between 1990 and 2019 for T2DM ASPRs in males and females increased in all 27 EU countries and the UK. These percentage change data are displayed in Supplementary Tables 1a and 1b. The largest increases in T2DM ASPR between 1990 and 2019 were observed in Luxembourg, Ireland and the UK for both males and females. (Luxembourg: males + 269.1%, females + 219.2%; Ireland: males + 191.9%, females + 165.7%; UK: males + 128.6%, females + 114.6%).

### Trends in T2DM disability-adjusted life years

Relative reductions in DALYs were observed in 3 of 28 countries for males (Cyprus, Malta and the Netherlands), and 11 of 28 countries for females. The greatest relative increase in DALYs was observed in Luxembourg for males (+ 99.3%), and the UK for females (+ 35.9%). The largest relative reduction in DALYs was observed in Cyprus for both sexes (− 26.2% for males, − 31.2% for females). Data are presented in Supplementary Tables 1a and 1b.

### Joinpoint analysis for T2DM mortality

**Figure 2:**
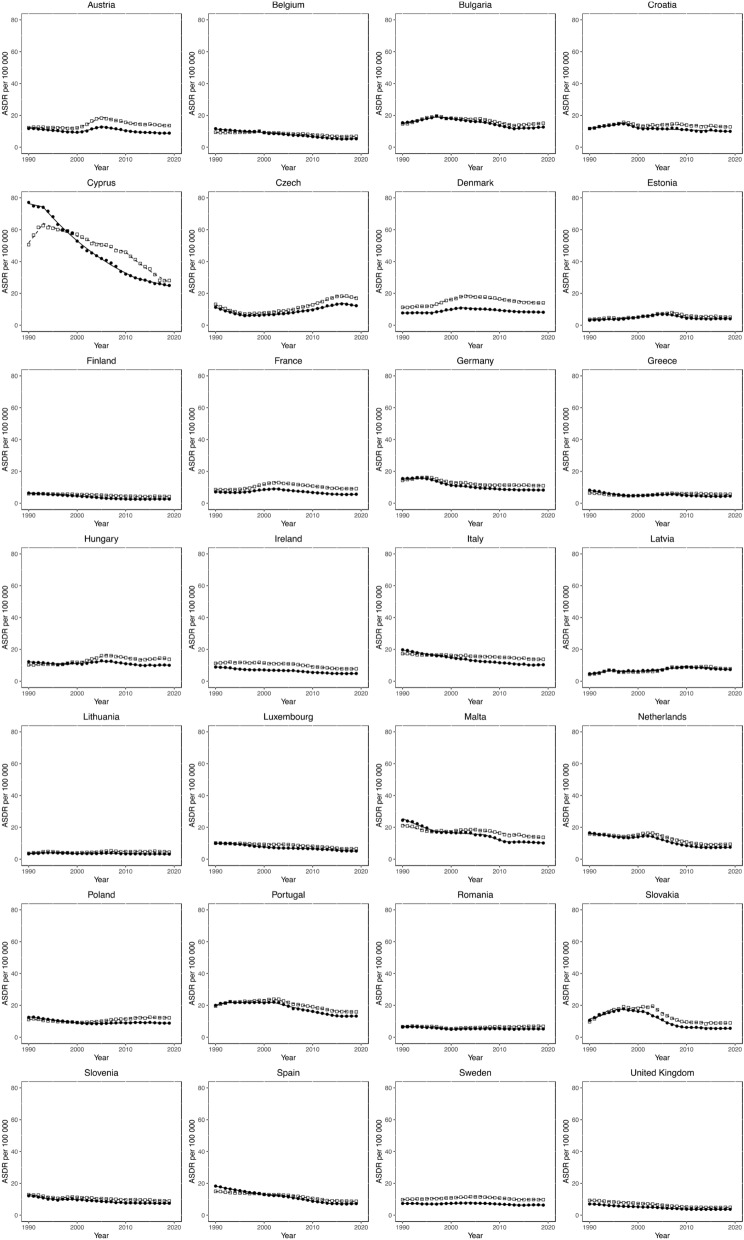
Trends in T2DM mortality. Trends in age-standardised death rates (ASDRs) per 100,000 population resulting directly from type 2 diabetes mellitus (T2DM) in all European Union (EU) countries and the UK from 1990 to 2019. Open squares represent males, filled circles represent females.

Sex-specific Joinpoint regression analyses for T2DM ASMRs between 1990 and 2019 are displayed in Fig. 2, and Tables 1 and 2. Significant ASMR estimated annual percentage changes (EAPCs) for each trend are presented (*p* value < 0.05). Trends in ASMRs varied both between and within countries. The single greatest increase in ASMR was observed in Slovenia for both sexes (EAPC + 14.4% for males between 1990 and 1994 and + 7.6% for females between 1990 and 1996). Slovenia also reported the single greatest reductions in ASMRs in both sexes (EAPC − 11.1% for males between 2003 and 2009 and − 12.2% for females between 2002 and 2009). No country observed persistently positive trends, for either sex. Persistently negative Joinpoint trends between 1990 and 2019 in ASMR were observed in Finland, Italy, Luxembourg and Spain for males, and in Cyprus, Ireland, Luxembourg, Malta and Spain for females. For both sexes, data for the UK demonstrates significant decreases in ASMR EAPCs from 1990 to 2011, followed by a plateau to insignificant EAPCs observed until 2019.

**Table 1:** Joinpoint analysis for age-standardised mortality rates (ASMRs) per 100,000 population resulting directly from type 2 diabetes mellitus (T2DM) in males.

	Trend 1		Trend 2		Trend 3		Trend 4	
	Years	EAPC	Years	EAPC	Years	EAPC	Years	EAPC
Austria	1990–2000	− 0.4* *(−0.7* to *−* *0.1)*	2000–2005	+ 9.5* *(*+ *8.3* to + *10.8)*	2005–2011	−3.9* *(−* *4.6* to *−* *3.1)*	2011–2019	−1.2* *(−1.6* to−*0.8)*
Belgium	1990–1999	+ 0.4* *(*+ *0.1* to + *0.8)*	1999–2009	−1.7* *(−2.0* to*−* *1.4)*	2009–2016	−2.7* *(−3.3* to *−* *2.1)*	2016–2019	+ 1.0 *(−* *0.8* to + *2.9)*
Bulgaria	1990–1996	+ 4.8* *(*+ *4.0* to + *5.7)*	1996–2007	−0.9* *(− 1.3* to *− 0.6)*	2007–2013	− 3.8* *(− 4.9 *to *− 2.8)*	2013–2019	+ 1.7* *(*+ *0.9* to + *2.5)*
Croatia	1990–1997	+ 4.2* *(*+ *3.3* to + *5.1)*	1997–2001	− 3.9* *(− 7.1 *to *− 0.6)*	2001–2007	+ 1.3 *(− 0.2* to + *2.8)*	2007–2019	− 1.1* *(− 1.5* to *− 0.7)*
Cyprus	1990–1993	+ 7.3* *(*+ *3.5* to + *11.3)*	1993–2010	− 1.9* *(− 2.2* to *− 1.7)*	2010–2019	− 5.8* *(− 6.4 *to *− 5.2)*		
Czech Republic	1990–1996	− 9.9* *(− 10.8* to *− 9.1)*	1996–2005	+ 3.4* *(*+ *2.8* to + *4.0)*	2005–2015	+ 7.1* *(*+ *6.5* to + *7.6)*	2015–2019	− 1.5 *(− 3.2* to + *0.2)*
Denmark	1990–1996	+ 1.3* *(*+ *0.3* to + *2.4)*	1996–1999	+ 8.5* *(*+ *2.2* to + *15.1)*	1999–2003	+ 4.7* *(*+ *1.7* to + *7.9)*	2003–2019	− 1.9* *(− 2.1* to *− 1.7)*
Estonia	1990–1999	+ 2.4* *(*+ *1.0* to + *3.9)*	1999–2007	+ 7.0* *(*+ *4.8* to + *9.3)*	2007–2010	− 10.9 *(− 23.7* to + *4.1)*	2010–2019	− 1.5* *(− 2.9* to *− 0.1)*
Finland	1990–2002	− 0.9* *(− 1.1* to *− 0.7)*	2002–2012	− 2.4* *(− 2.7* to *− 2.1)*	2012–2019	− 0.1 *(− 0.6* to + *0.4)*		
France	1990–1995	+ 0.4 *(− 0.4* to + *1.3)*	1995–2002	+ 6.4* *(*+ *5.7* to + *7.0)*	2002–2016	− 2.7* *(− 2.9 *to *− 2.5)*	2016–2019	0.0 *(− 1.8* to + *1.9)*
Germany	1990–1995	+ 2.9* *(*+ *2.0* to + *3.8)*	1995–1999	− 4.9* *(− 6.7* to *− 3.0)*	1999–2007	− 2.2* *(− 2.7* to *− 1.6)*	2007–2019	− 0.2 *(− 0.4* to *0.0)*
Greece	1990–1998	− 4.4* *(− 5.0* to *− 3.9)*	1998–2001	+ 2.1 *(− 2.8* to + *7.2)*	2001–2007	+ 4.3* *(*+ *3.1* to + *5.4)*	2007–2019	− 0.9* *(− 1.2* to *− 0.7)*
Hungary	1990–2000	+ 1.3* *(*+ *0.7* to + *1.9)*	2000–2006	+ 6.2* *(*+ *4.3* to + *8.1)*	2006–2013	− 2.8* *(− 4.1 to − 1.5)*	2013–2019	+ 0.9 *(− 0.4* to + *2.3)*
Ireland	1990–1993	+ 2.1 *(− 0.4* to + *4.6)*	1993–2006	− 0.8* *(− 1.1* to *− 0.5)*	2006–2014	− 3.7* *(− 4.3* to *− 3.1)*	2014–2019	− 0.7 *(− 1.8* to + *0.5)*
Italy	1990–2009	− 0.6* *(− 0.7* to *− 0.5)*	2009–2019	− 1.1* *(− 1.4* to *− 0.9)*				
Latvia	1990–1994	+ 12.2* (+ 6.7 to + 17.9)	1994–2000	− 3.0 (− 6.3 to + 0.5)	2000–2010	+ 5.5* (+ 4.0 to + 7.0)	2010–2019	− 1.8* (− 3.2 to − 0.4)
Lithuania	1990–1994	+ 6.9* *(*+ *3.3* to + *10.5)*	1994–1999	− 4.6* *(− 7.8* to *− 1.4)*	1999–2006	+ 3.8* *(*+ *2.0* to + *5.7)*	2006–2019	− 0.5 *(− 1.0* to + *0.1*
Luxembourg	1990–2005	− 0.7* (− 0.9 to − 0.5)	2005–2013	− 2.3* (− 2.9 to − 1.8)	2013–2016	− 4.2* (− 8.0 to − 0.3)	2016–2019	− 0.3 (− 2.3 to + 1.7)
Malta	1990–1996	− 3.7* (− 4.9 to − 2.5)	1996–2005	+ 0.8 (0.0 to + 1.6)	2005–2019	− 2.3* (− 2.6 to − 1.9)		
Netherlands	1990–1997	− 1.6* *(− 2.0* to *− 1.2)*	1997–2003	+ 2.3* *(*+ *1.6* to + *3.0)*	2003–2013	− 5.7* *(− 6.0* to *− 5.5)*	2013–2019	+ 0.5 *(− 0.1* to + *1.0)*
Poland	1990–2001	− 2.0* *(− 2.4* to *− 1.6)*	2001–2012	+ 2.7* *(*+ *2.3* to + *3.2)*	2012–2019	+ 0.1 *(− 0.7* to + *0.8)*		
Portugal	1990–1992	+ 4.8* (+ 0.9 to + 8.8)	1992–2002	+ 1.0* (+ 0.7 to + 1.4)	2002–2015	− 2.9* (− 3.1 to − 2.7)	2015– 2019	− 0.7 (− 1.9 to + 0.5)
Romania	1990–1996	+ 0.2 (− 0.8 to + 1.2)	1996–2000	− 6.0* (− 8.7 to − 3.1)	2000–2003	+ 3.9 (− 2.1 to + 10.3)	2003–2019	+ 0.9* (+ 0.7 to + 1.2)
Slovakia	1990–1996	− 3.4* (− 4.2 to − 2.6)	1996–1999	+ 2.5 (− 2.5 to + 7.7)	1999–2006	− 1.7* (− 2.5 to − 0.8)	2006–2019	− 1.0* (− 1.2 to − 0.7)
Slovenia	1990–1994	+ 14.4* (+ 11.1 to + 17.9)	1994–2003	+ 1.3* (+ 0.2 to + 2.3)	2003–2009	− 11.1* (− 13.0 to − 9.2)	2009–2019	− 0.8* (− 1.5 to − 0.1)
Spain	1990–2006	− 1.2* (− 1.3 to − 1.1)	2006–2014	− 3.8* (− 4.1 to − 3.4)	2014–2019	− 0.8* (− 1.4 to − 0.1)		
Sweden	1990–2006	+ 1.1* (+ 1.0 to + 1.2)	2006–2014	− 2.2* (− 2.6 to − 1.7)	2014–2019	− 0.1 (− 0.8 to + 0.6)		
United Kingdom	1990–2003	− 2.4* *(− 2.5* to *− 2.2)*	2003–2011	− 4.1* *(− 4.5* to *− 3.8)*	2011–2019	*0.0* *(− 0.3* to + *0.3)*		

Italic values indicates 95% confidence intervals.

EAPC indicates estimated annual percentage change with 95% confidence intervals in brackets.

*Significantly different from 0 (P < 0.05).

**Table 2:** Joinpoint analysis for age-standardised mortality rates (ASMRs) per 100,000 population resulting directly from type 2 diabetes mellitus (T2DM) in females.

	Trend 1		Trend 2		Trend 3		Trend 4	
	Years	EAPC	Years	EAPC	Years	EAPC	Years	EAPC
Austria	1990–2000	− 2.6* *(− 2.9* to *− 2.3)*	2000–2005	+ 7.1* *(*+ *5.8* to + *8.4)*	2005–2012	− 4.2* *(− 4.8* to *− 3.6)*	2012–2019	− 1.3* *(− 1.8* to *− 0.8)*
Belgium	1990–1999	− 2.0* *(− 2.4* to − 1.6)	1999–2009	− 3.1* *(− 3.5* to *− 2.8)*	2009–2016	− 4.3* *(− 5.0* to *− 3.7)*	2016–2019	+ 1.8 *(− 0.3* to + *3.9)*
Bulgaria	1990–1997	+ 3.3* *(*+ *2.9* to + *3.8)*	1997–2008	− 2.1* *(− 2.4* to *− 1.9)*	2008–2013	− 5.0* *(− 6.0* to *− 4.0)*	2013–2019	+ 1.3* *(*+ *0.7* to + *1.8)*
Croatia	1990–1997	+ 3.3* *(*+ *2.0* to + *4.7)*	1997–2000	− 7.0 *(− 15.8* to + *2.8)*	2000–2019	− 1.0* *(− 1.3* to *− 0.7)*		
Cyprus	1990–1993	− 0.9 *(− 3.7* to + *1.9)*	1993–2012	− 4.7* *(− 4.9* to *− 4.5)*	2012–2019	− 2.6* *(− 3.4* to *− 1.9)*		
Czech Republic	1990–1996	− 10.2* *(− 11.0 *to *− 9.3)*	1996–2004	+ 2.4* *(*+ *1.7* to + *3.2)*	2004–2016	+ 5.6* *(*+ *5.2* to + *6.0)*	2016–2019	− 3.9* *(− 6.5* to *− 1.2)*
Denmark	1990–1996	+ 0.3 *(− 0.6* to + *1.1)*	1996–2002	+ 5.9* *(*+ *4.8* to + *7.0)*	2002–2015	− 2.1* *(− 2.4* to *− 1.8)*	2015–2019	− 0.6 *(− 2.1* to + *0.9)*
Estonia	1990–1997	+ 3.2* *(*+ *1.6* to + *4.8)*	1997–2005	+ 7.9* *(*+ *6.3* to + *9.6)*	2005–2012	− 8.3* *(− 10.0* to *− 6.5)*	2012–2019	+ 0.2 *(− 1.3* to + *1.7)*
Finland	1990–2002	− 3.6* *(− 3.9* to *− 3.4)*	2002–2006	− 7.0* *(− 8.8 to − 5.2)*	2006–2011	− 3.9* *(− 5.1* to *− 2.7)*	2011–2019	+ 0.4 *(0.0* to + *0.8)*
France	1990–1994	− 2.0* *(− 3.2* to *− 0.9)*	1994–2002	+ 4.2* *(*+ *3.7* to + *4.7)*	2002–2015	− 3.7* *(− 4.0* to *− 3.5)*	2015–2019	+ 0.1 *(− 1.1* to + *1.2)*
Germany	1990–1995	0.0 *(− 0.8* to + *0.8)*	1995–2000	− 6.5* *(− 7.5* to *− 5.4)*	2000–2011	− 2.4* *(− 2.7* to *− 2.2)*	2011–2019	− 0.5* *(− 0.9* to *− 0.1)*
Greece	1990–1998	− 6.8* *(− 7.4* to *− 6.2)*	1998–2007	+ 1.9* *(*+ *1.2* to + *2.6)*	2007–2014	− 3.6* *(− 4.6* to *− 2.6)*	2014–2019	+ 0.5 *(− 0.9* to + *1.8)*
Hungary	1990–1997	− 2.1* *(− 3.1* to *− 1.0)*	1997–2006	− 2.0* *(*+ *1.2* to + *2.9)*	2006–2013	− 3.3* *(− 4.6* to *− 2.0)*	2013–2019	+ 0.2 *(− 1.1* to + *1.5)*
Ireland	1990–1996	− 3.4* *(− 4.1* to *− 2.7)*	1996–2006	− 1.2* *(− 1.5* to *− 0.8)*	2006–2014	− 3.7* *(− 4.2* to *− 3.1)*	2014–2019	− 0.2 *(− 1.2* to + *0.7)*
Italy	1990–2006	− 2.8* *(− 2.9* to *− 2.6)*	2006–2017	− 1.9* *(− 2.2* to *− 1.7)*	2017–2019	+ 1.3 *(− 2.1* to + *4.8)*		
Latvia	1990− 1994	9.4* (+ 5.5 to + 13.5)	1994–2000	− 1.0 (− 3.5 to + 1.6)	2000–2010	+ 3.8* (+ 2.7 to + 4.9)	2010–2019	− 2.5* (− 3.5 to − 1.4)
Lithuania	1990–1994	+ 4.7* *(*+ *1.5* to + *8.0)*	1994–2002	− 2.0* *(− 3.3* to *− 0.7)*	2002–2006	+ 1.9 *(− 3.0* to + *7.0)*	2006–2019	− 1.4* *(− 1.9* to *− 0.9)*
Luxembourg	1990–1993	− 0.2 (− 3.0 to + 2.6)	1993–2002	− 3.6* (− 4.2 to − 3.0)	2002–2010	− 1.0* (− 1.7 to − 0.2)	2010–2019	− 3.1* (− 3.6 to − 2.6)
Malta	1990–1997	− 5.4* (− 6.4 to − 4.4)	1997–2007	− 1.2* (− 1.9 to − 0.5)	2007–2011	− 7.9* (− 11.5 to − 4.2)	2011–2019	− 0.9 (− 1.7 – 0.0)
Netherlands	1990–1998	− 2.7* *(− 3.2 *to *− 2.3)*	1998–2002	+ 3.1* *(*+ *1.0* to + *5.1)*	2002–2012	− 6.7* *(− 7.0* to *− 6.3)*	2012–2019	− 0.1 *(− 0.6* to + *0.4)*
Poland	1990–2002	− 3.5* *(− 3.7* to *− 3.2)*	2002–2013	+ 0.8* *(*+ *0.5* to + *1.1)*	2013–2019	− 0.8* *(− 1.5* to *− 0.1)*		
Portugal	1990–1992	+ 4.1 (− 0.8 to + 9.2)	1992–2002	0.0 (− 0.4 to + 0.5)	2002–2015	− 3.7* (− 4.0 to − 3.5)	2015–2019	− 0.1 (− 1.6 to + 1.4)
Romania	1990–1993	+ 0.7 (− 1.7 to + 3.1)	1993–2000	− 3.8* (− 4.5 to − 3.0)	2000–2005	+ 1.0 (− 0.5 to + 2.5)	2005–2019	− 0.2 (− 0.4 to + 0.1)
Slovakia	1990–1996	− 4.0* (− 4.7 to − 3.4)	1996–1999	+ 1.6 (− 2.4 to + 5.8)	1999–2010	− 2.4* (− 2.7 to − 2.1)	2010–2019	− 0.3 (− 0.7 – 0.0)
Slovenia	1990–1996	+ 7.6* (+ 5.6 to + 9.7)	1996–2002	− 2.2 (− 4.6 to + 0.3)	2002–2009	− 12.2* (− 13.9 to − 10.5)	2009–2019	− 1.5* (− 2.4 to − 0.7)
Spain	1990–2001	− 3.3* (− 3.5 to − 3.1)	2001–2005	− 2.1* (− 3.8 to − 0.4)	2005–2014	− 5.2* (− 5.5 to − 4.8)	2014–2019	− 0.1 (− 0.9 to + 0.7)
Sweden	1990–1996	− 1.1* (− 1.8 to − 0.4)	1996–2004	+ 1.3* (+ 0.8 to + 1.9)	2004–2014	− 2.0* (− 2.3 to − 1.6)	2014–2019	+ 0.2 (− 0.8 to + 1.1)
United Kingdom	1990–1997	− 3.5* *(− 3.8* to *− 3.2)*	1997–2003	− 2.0* *(− 2.4* to *− 1.5)*	2003–2011	− 3.6* *(− 3.9* to *− 3.3)*	2011–2019	+ 0.1 *(− 0.1* to + *0.4)*

Italic values indicates 95% confidence intervals.

EAPC indicates estimated annual percentage change with 95% confidence intervals in brackets.

*Significantly different from 0 (P < 0.05).

### Joinpoint analysis for T2DM prevalence

**Figure 3:**
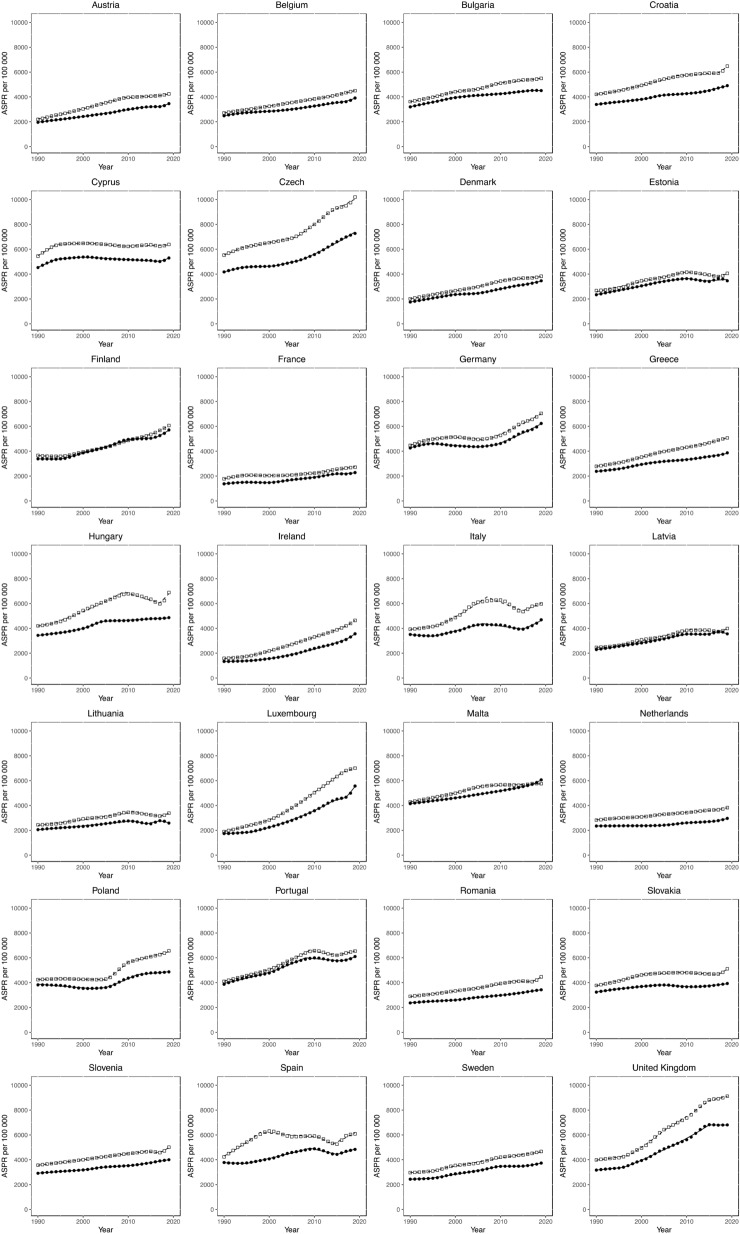
Trends in T2DM prevalence. Trends in age-standardised prevalence rates (ASPRs) per 100,000 population for type 2 diabetes mellitus (T2DM) in all European Union (EU) countries and the UK from 1990 to 2019. Open squares represent males, filled circles represent females.

Sex-specific Joinpoint regression analyses for T2DM ASPRs between 1990 and 2019 are displayed in Fig. 3, and Tables 3 and 4. Significant ASPR EAPCs are presented (*p* value < 0.05). Increasing trends in T2DM prevalence were observed in all countries for both sexes. The rate of increase in ASPR varied across and within countries. Consistent, significantly positive trends were observed in 12 of the 28 countries in males, and in 11 countries in females. Over the time period studied, the most rapidly increasing ASPRs in males were observed in Poland (2005–2010 EAPC + 6%), Hungary (2017–2019 EAPC + 6%) and Luxembourg (2000–2010 EAPC + 6%). For females, the most rapidly increasing ASPRs for T2DM were observed between 2017 and 2019 in Ireland (EAPC + 6.8%) and Luxembourg (EAPC + 8.8%). The UK observed consistent, significantly positive increases in T2DM ASPRs from 1996/7–2015 for both males and females (EAPC increases of + 3.2% to + 5.0%), followed by a plateau from 2015, where non-significant EAPCs of between 0 and + 0.9% have been observed. Only for females in Latvia were significant EAPC decreases identified over the time periods covered by the most recent trends (2017–2019 EAPC − 2.1%). No country displayed persistently negative trends for either sex.

**Table 3:** Joinpoint analysis for age-standardised prevalence rates (ASPRs) per 100,000 population for type 2 diabetes (T2DM) in males.

	Trend 1		Trend 2		Trend 3		Trend 4	
	Years	EAPC	Years	EAPC	Years	EAPC	Years	EAPC
Austria	1990–2003	+ 3.2* *(*+ *3.1* to + *3.3)*	2003–2009	+ 2.7* *(*+ *2.5* to + *3.0)*	2009–2017	+ 0.6* *(*+ *0.4* to + *0.7)*	2017–2019	+ 1.6* *(*+ *0.4* to + *2.8)*
Belgium	1990–1993	+ 2.1* *(*+ *1.8* to + *2.3)*	1993–2005	+ 1.8* *(*+ *1.7* to + *1.8)*	2005–2014	+ 1.6* *(*+ *1.5* to + *1.6)*	2014–2019	+ 2.0* *(*+ *1.9* to + *2.1)*
Bulgaria	1990–2000	+ 2.0* *(*+ *2.0* to + *2.1)*	2000–2005	+ 0.9* *(*+ *0.6* to + *1.3)*	2005–2010	+ 2.1* *(*+ *1.8* to + *2.5)*	2010–2019	+ 0.8* *(*+ *0.7* to + *0.9)*
Croatia	1990–1994	+ 1.2* *(*+ *0.9* to + *1.6)*	1994–2008	+ 1.8* *(*+ *1.8* to + *1.9)*	2008–2017	+ 0.4* *(*+ *0.3* to + *0.5)*	2017–2019	+ 4.1* *(*+ *3.0* to + *5.2)*
Cyprus	1990–1994	+ 3.9* *(*+ *3.4* to + *4.4)*	1994–2001	+ 0.3 *(0.0* to + *0.5)*	2001–2009	− 0.5* *(− 0.7* to *− 0.3)*	2009–2019	+ 0.1* *(0.0* to + *0.3)*
Czech Republic	1990–1994	+ 2.5* *(*+ *1.9* to + *3.1)*	1994–2006	+ 1.1* *(*+ *1.0* to + *1.2)*	2006–2013	+ 3.5* *(*+ *3.2* to + *3.9)*	2013–2019	+ 2.0* *(*+ *1.7* to + *2.4)*
Denmark	1990–1996	+ 3.1* *(*+ *2.9* to + *3.3)*	1996–2004	+ 2.3* *(*+ *2.1* to + *2.5)*	2004–2011	+ 2.8* *(*+ *2.6* to + *3.0)*	2011–2019	+ 0.9* *(*+ *0.8* to + *1.0)*
Estonia	1990–2001	+ 2.8* *(*+ *2.5* to + *3.1)*	2001–2010	+ 2.0* *(*+ *1.5* to + *2.5)*	2010–2017	− 1.3* *(− 2.0 to − 0.6)*	2017–2019	+ 2.9 *(− 1.4* to + *7.5)*
Finland	1990–1993	− 0.7 *(− 1.4* to + *0.1)*	1993–1996	+ 0.3 *(− 1.2* to + *1.8)*	1996–2016	+ 2.1* *(*+ *2.1* to + *2.2)*	2016–2019	+ 3.4* *(*+ *2.6* to + *4.2)*
France	1990–1994	+ 3.6* *(*+ *2.9* to + *4.4)*	1994–2003	− 0.2 *(− 0.4* to + *0.1)*	2003–2010	+ 1.4* *(*+ *1.0* to + *1.8)*	2010–2019	+ 2.3* *(*+ *2.1* to + *2.5)*
Germany	1990–1994	+ 2.4* *(*+ *1.6* to + *3.2)*	1994–1999	+ 0.9* *(*+ *0.1* to + *1.7)*	1999–2007	− 0.6* *(− 0.9 to − 0.3)*	2007–2019	+ 3.0* *(*+ *2.9* to + *3.2)*
Greece	1990–1995	+ 2.0* *(*+ *1.9* to + *2.1)*	1995–2004	+ 2.7* *(*+ *2.7* to + *2.8)*	2004–2014	+ 1.6* *(*+ *1.5* to + *1.6)*	2014–2019	+ 2.1* *(*+ *1.9* to + *2.2)*
Hungary	1990–1993	+ 1.4 *(− 0.4* to + *3.1)*	1993–2009	+ 2.9* *(*+ *2.8* to + *3.1)*	2009–2017	− 1.7* *(− 2.2* to − 1.3)	2017–2019	+ 6.0* *(*+ *2.4* to + *9.7)*
Ireland	1990–1995	+ 2.0* *(*+ *1.7* to + *2.3)*	1995–2007	+ 4.6* *(*+ *4.5* to + *4.7)*	2007–2017	+ 3.5* *(*+ *3.4* to + *3.6)*	2017–2019	+ 5.3* *(*+ *4.0* to + *6.6)*
Italy	1990–1996	+ 1.2* *(*+ *0.3* to + *2.1)*	1996–2007	+ 4.1* *(*+ *3.7* to + *4.5)*	2007–2015	− 2.3* *(− 2.9* to *− 1.6)*	2015–2019	+ 2.6* *(*+ *0.9* to + *4.3)*
Latvia	1990–1993	+ 1.1 *(− 0.8* to + *2.9)*	1993–2011	+ 2.4* *(*+ *2.3* to + *2.6)*	2011–2017	− 0.7 *(− 1.5* to + *0.1)*	2017–2019	+ 2.8 *(− 0.9* to + *6.7)*
Lithuania	1990–2011	+ 1.8* *(*+ *1.7* to + *1.9)*	2011–2017	− 1.8* *(− 2.6* to *− 0.9)*	2017–2019	+ 3.8 *(− 0.2* to + *8.0)*		
Luxembourg	1990–2000	+ 4.0* *(*+ *3.9* to + *4.1)*	2000–2010	+ 6.0* *(*+ *5.9* to + *6.1)*	2010–2016	+ 4.6* *(*+ *4.3* to + *4.9)*	2016–2019	+ 1.9* (+ 1.3 to + 2.6)
Malta	1990–1999	+ 1.5* *(*+ *1.4* to + *1.7)*	1999–2006	+ 1.8* *(*+ *1.6* to + *2.0)*	2006–2019	+ 0.2* *(*+ *0.2* to + *0.3)*		
Netherlands	1990–2001	+ 0.8* *(*+ *0.7* to + *0.9)*	2001–2004	+ 1.4* *(*+ *0.1* to + *2.8)*	2004–2017	+ 0.9* *(*+ *0.9* to + *1.0)*	2017–2019	+ 1.9* *(*+ *0.5* to + *3.3)*
Poland	1990–1995	+ 0.3* *(*+ *0.1* to + *0.5)*	1995–2005	− 0.2* *(− 0.3* to *− 0.1)*	2005–2010	+ 6.0* *(*+ *5.6* to + *6.3)*	2010–2019	+ 1.5* *(*+ *1.4* to + *1.6)*
Portugal	1990–2000	+ 2.1* *(*+ *2.0* to + *2.1)*	2000–2009	+ 3.0* *(*+ *2.9* to + *3.1)*	2009–2015	− 1.0* *(− 1.2* to *− 0.9)*	2015–2019	+ 1.3* *(*+ *1.1* to + *1.6)*
Romania	1990–2004	+ 1.4* *(*+ *1.4* to + *1.5)*	2004–2013	+ 1.8* *(*+ *1.6* to + *1.9)*	2013–2017	− 0.2 *(− 0.9* to + *0.4)*	2017–2019	+ 4.3* *(*+ *2.9* to + *5.6)*
Slovakia	1990–2008	+ 1.2* *(*+ *1.2* to + *1.2)*	2008–2014	+ 1.0* *(*+ *0.9* to + *1.0)*	2014–2017	− 0.7* *(− 1.0* to *− 0.4)*	2017–2019	+ 4.3* *(*+ *4.0* to + *4.7)*
Slovenia	1990–2001	+ 2.1* *(*+ *2.0* to + *2.2)*	2001–2009	+ 0.3* *(*+ *0.2* to + *0.5)*	2009–2017	− 0.4* *(− 0.6* to *− 0.3)*	2017–2019	+ 4.5* *(*+ *3.3* to + *5.7)*
Spain	1990–1998	+ 4.7* *(*+ *4.1* to + *5.3)*	1998–2011	− 0.5* *(− 0.8* to *− 0.2)*	2011–2014	− 3.3 *(− 7.9* to + *1.6)*	2014–2019	+ 3.3* *(*+ *2.1* to + *4.4)*
Sweden	1990–1995	+ 0.8* *(*+ *0.1* to + *1.4)*	1995–1998	+ 3.0* (+ 0.2 to + 6.0)	1998–2010	+ 1.8* (+ 1.6 to + 2.0)	2010–2019	+ 1.2* (+ 0.9 to + 1.5)
United Kingdom	1990–1997	+ 1.2* *(*+ *0.8* to + *1.6)*	1997–2005	+ 5.0* *(*+ *4.6* to + *5.4)*	2005–2015	+ 3.2* *(*+ *3.0* to + *3.5)*	2015–2019	+ *0.9* *(0.0* to + *1.8)*

Italic values indicates 95% confidence intervals.

Data presented as estimated annual percentage change (EAPC %), with 95% confidence intervals in brackets. P values deemed significant if < 0.05.

**Table 4:** Joinpoint analysis for age-standardised prevalence rates (ASPRs) per 100,000 population for type 2 diabetes (T2DM) in females.

	Trend 1		Trend 2		Trend 3		Trend 4	
	Years	EAPC	Years	EAPC	Years	EAPC	Years	EAPC
Austria	1990–2013	+ 2.1* *(*+ *2.0* to + *2.1)*	2013–2017	+ 0.4 *(− 0.3* to + *1.2)*	2017–2019	+ 3.4* *(*+ *1.9* to + *4.9)*		
Belgium	1990–1994	+ 2.2* *(*+ *1.9* to + *2.5)*	1994–2003	+ 0.8* *(*+ *0.7* to + *0.9)*	2003–2017	+ 1.6* *(*+ *1.6* to + *1.7)*	2017–2019	+ 3.2* *(*+ *2.3* to + *4.1)*
Bulgaria	1990–2000	+ 2.1* *(*+ *2.1* to + *2.2)*	2000–2011	+ 0.7* *(*+ *0.6* to + *0.8)*	2011–2017	+ 0.9* *(*+ *0.7* to + *1.1)*	2017–2019	− 0.2 *(− 1.0* to + *0.6)*
Croatia	1990–2001	+ 1.1* *(*+ *1.1* to + *1.2)*	2001–2004	+ 2.2* *(*+ *1.6* to + *2.8)*	2004–2013	+ 0.6* *(*+ *0.6* to + *0.7)*	2013–2019	+ 2.0* *(*+ *1.9* to + *2.1)*
Cyprus	1990–1994	+ 3.4* *(*+ *3.0* to + *3.7)*	1994–2000	+ 0.6* *(*+ *0.3* to + *0.8)*	2000–2017	− 0.4* *(− 0.4* to* − 0.4)*	2017–2019	+ 2.5* *(*+ *1.4* to + *3.5)*
Czech Republic	1990–1994	+ 2.1* *(*+ *1.5* to + *2.7)*	1994–2001	+ 0.3 *(0.0* to + *0.6)*	2001–2007	+ 1.6* *(*+ *1.2* to + *2.1)*	2007–2019	+ 3.1* *(*+ *3.0* to + *3.2)*
Denmark	1990–1999	+ 3.2* *(*+ *3.0* to + *3.3)*	1999–2005	+ 0.8* *(*+ *0.5* to + *1.2)*	2005–2012	+ 2.7* *(*+ *2.5* to + *3.0)*	2012–2019	+ 2.1* *(*+ *1.9* to + *2.3)*
Estonia	1990–2004	+ 2.6* *(*+ *2.4* to + *2.8)*	2004–2010	+ 1.3* *(*+ *0.5* to + *2.2)*	2010–2014	− 1.5 *(− 3.3* to + *0.4)*	2014–2019	+ 0.7 *(− 0.1* to + *1.6)*
Finland	1990–1995	− 0.1 *(− 0.5* to + *0.3)*	1995–2010	+ 2.6* *(*+ *2.5* to + *2.7)*	2010–2016	+ 0.4 *(0.0* to + *0.8)*	2016–2019	+ 3.9* *(*+ *3.0* to + *4.8)*
France	1990–1994	+ 2.2* *(*+ *1.2* to + *3.2)*	1994–2000	− 0.4 *(− 1.0* to + *0.3)*	2000–2014	+ 2.7* *(*+ *2.5* to + *2.9)*	2014–2019	+ 1.1* *(*+ *0.4* to + *1.7)*
Germany	1990–1994	+ 2.0* *(*+ *1.2* to + *2.7)*	1994–2005	− 0.6* *(− 0.8* to *− 0.4)*	2005–2009	+ 1.0 *(− 0.2* to + *2.2)*	2009–2019	+ 3.2* *(*+ *3.0* to + *3.4)*
Greece	1990–1995	+ 1.7* *(*+ *1.4* to + *2.0)*	1995–2002	+ 2.6* *(*+ *2.3* to + *2.8)*	2002–2012	+ 1.0* *(*+ *0.9* to + *1.1)*	2012–2019	+ 1.7* *(*+ *1.5* to + *1.9)*
Hungary	1990–1996	+ 1.2* *(*+ *1.0* to + *1.5)*	1996–2000	+ 1.9* *(*+ *1.3* to + *2.5)*	2000–2004	+ 3.4* *(*+ *2.7* to + *4.0)*	2004–2019	+ 0.4* *(*+ *0.4* to + *0.5)*
Ireland	1990–1996	+ 0.8* *(*+ *0.2* to + *1.3)*	1996–2001	+ 2.9* *(*+ *1.9* to + *4.0)*	2001–2017	+ 4.2* *(*+ *4.0* to + *4.3)*	2017–2019	+ 6.8* *(*+ *3.4* to + *10.4)*
Italy	1990–1995	− 0.8* *(− 1.4* to *− 0.2)*	1995–2006	+ 2.5* *(*+ *2.2* to + *2.7)*	2006–2015	− 1.2* *(− 1.5* to *− 0.9)*	2015–2019	+ 4.1* *(*+ *3.2* to + *5.0)*
Latvia	1990–2010	+ 2.2* *(*+ *2.2* to + *2.3)*	2010–2014	− 0.2 *(− 1.2* to + *0.7)*	2014–2017	+ 2.1* *(*+ *0.1* to + *4.2)*	2017–2019	− 2.1* *(− 4.0 *to *− 0.1)*
Lithuania	1990–2011	+ 1.5* *(*+ *1.4* to + *1.5)*	2011–2014	− 3.2 *(− 6.5* to + *0.3)*	2014–2017	+ 3.2 *(− 0.3* to + *6.9)*	2017–2019	− 2.8 *(− 6.1* to + *0.7)*
Luxembourg	1990–1996	+ 1.2* *(*+ *0.8* to + *1.6)*	1996–2014	+ 4.8* *(*+ *4.7* to + *4.9)*	2014–2017	+ 2.5* *(*+ *0.2* to + *4.9)*	2017–2019	+ 8.8* *(*+ *6.3* to + *11.3)*
Malta	1990–2000	+ 1.1* *(*+ *1.0* to + *1.1)*	2000–2013	+ 1.2* *(*+ *1.2* to + *1.2)*	2013–2017	+ 1.4* *(*+ *1.4* to + *1.5)*	2017–2019	+ 3.2* *(*+ *3.0* to + *3.4)*
Netherlands	1990–2004	+ 0.1* *(*+ *0.1* to + *0.1)*	2004–2010	+ 1.5* *(*+ *1.4* to + *1.6)*	2010–2016	+ 0.7* *(*+ *0.6* to + *0.8)*	2016–2019	+ 2.7* *(*+ *2.4* to + *2.9)*
Poland	1990–2002	− 0.8* *(− 1.0* to *− 0.6)*	2002–2005	+ 0.8 *(− 1.9* to + *3.5)*	2005–2012	+ 3.7* *(*+ *3.2* to + *4.2)*	2012–2019	+ 0.7* *(*+ *0.3* to + *1.1)*
Portugal	1990–2000	+ 2.0* *(*+ *1.8* to + *2.3)*	2000–2008	+ 2.8* *(*+ *2.4* to + *3.1)*	2008–2016	− 0.5* *(− 0.9* to *− 0.2)*	2016–2019	+ 1.8* *(*+ *0.5* to + *3.1)*
Romania	1990–2001	+ 0.9* *(*+ *0.8* to + *1.0)*	2001–2004	+ 1.9* *(*+ *0.9* to + *2.9)*	2004–2012	+ 1.2* *(*+ *1.0* to + *1.3)*	2012–2019	+ 1.7* *(*+ *1.6* to + *1.8)*
Slovakia	1990–2001	+ 0.9* *(*+ *0.8* to + *0.9)*	2001–2004	+ 1.8* *(*+ *1.1* to + *2.6)*	2004–2011	+ 0.6* *(*+ *0.5* to + *0.8)*	2011–2019	+ 1.5* *(*+ *1.5* to + *1.6)*
Slovenia	1990–1995	+ 1.6* *(*+ *1.3* to + *1.8)*	1995–2004	+ 1.0* *(*+ *0.8* to + *1.1)*	2004–2012	− 0.6* *(− 0.8* to *− 0.5)*	2012–2019	+ 1.0* *(*+ *0.9* to + *1.2)*
Spain	1990–1995	− 0.3 *(− 0.7* to + *0.2)*	1995–2009	+ 2.1* *(*+ *2.0* to + *2.2)*	2009–2015	− 1.7* *(− 2.2* to *− 1.3)*	2015–2019	+ 2.3* *(*+ *1.6* to + *2.9)*
Sweden	1990–1994	+ 0.5 *(− 0.2* to + *1.3)*	1994–2010	+ 2.1* *(*+ *2.0* to + *2.2)*	2010–2015	− 0.1 *(− 0.8* to + *0.7)*	2015–2019	+ 1.7* *(*+ *0.9* to + *2.5)*
United Kingdom	1990–1996	+ 1.1* *(*+ *0.6* to + *1.6)*	1996–2004	+ 4.0* *(*+ *3.6* to + *4.5)*	2004–2015	+ 3.5* *(*+ *3.3* to + *3.8)*	2015–2019	0.0 *(− 1.0* to + *0.9)*

Italic values indicates 95% confidence intervals.

Data presented as estimated annual percentage change (EAPC %), with 95% confidence intervals in brackets. P values deemed significant if < 0.05.

### Joinpoint analysis for T2DM disability-adjusted life-years

**Figure 4:**
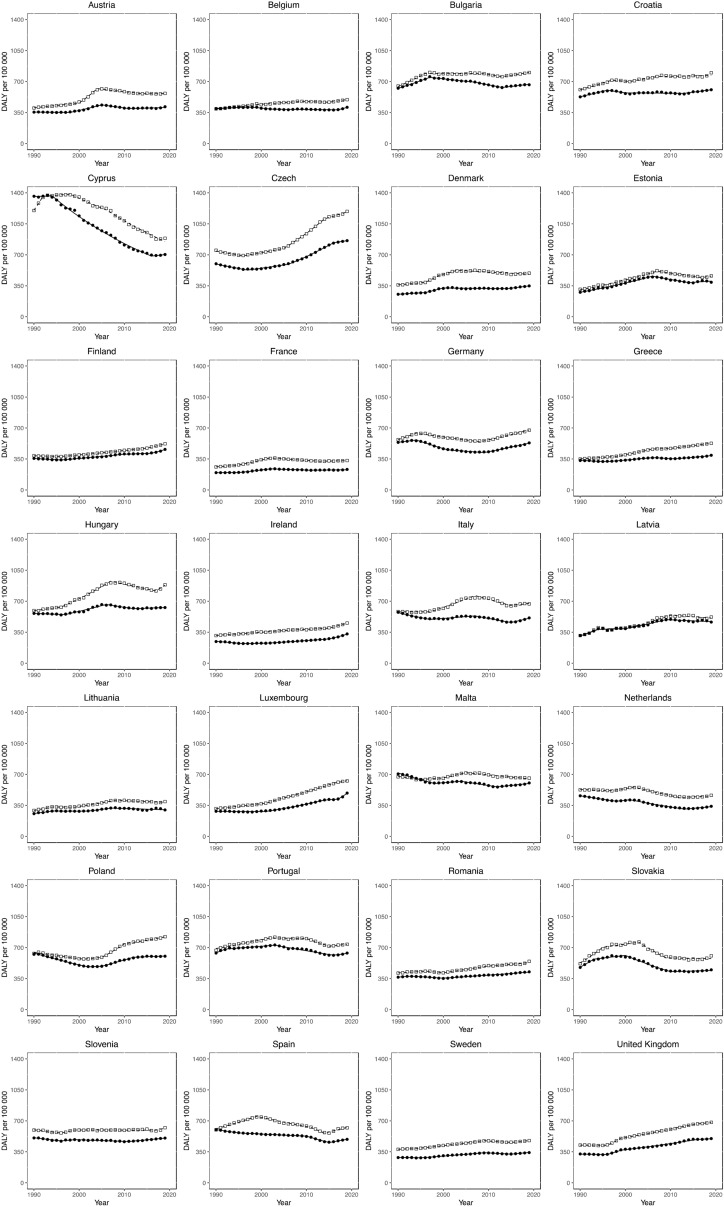
Trends in T2DM DALYs. Trends in age-standardised disability adjusted life years (DALYs) per 100,000 population for type 2 diabetes mellitus (T2DM) in all European Union (EU) countries and the UK from 1990 to 2019. Open squares represent males, filled circles represent females.

Sex-specific Joinpoint regression analyses for T2DM DALYs between 1990 and 2019 are displayed in Fig. 4 and Supplementary Tables 3 and 4. Significant DALY EAPCs are presented (*p* value < 0·05). No country demonstrated persistently negative trends. Persistent, significantly positive trends were observed only in Ireland for males. Notable rapid increases in DALY EAPCs were observed in the UK between 1996 and 1999 for males (EAPC + 5.9%) and females (EAPC + 4.8%). The most recent trends in the UK between (2015/16–2019) for both sexes demonstrate smaller increases in DALYs EAPCs to + 0.8% for males and + 0.5% for females.

## Discussion

In this observational analysis of trends in the disease burden of T2DM in EU countries, we identify increasing prevalence of T2DM across all 28 EU countries between 1990 and 2019, with the highest relative increases in T2DM prevalence observed in the UK, Luxembourg and Ireland for both sexes. Over the same 30-year study period, mortality trends generally decreased across EU countries, however, the trends were less uniform than those observed for prevalence.

Being aware that the present study is observational, we are cautious to not attribute causality to the identified trends, however probable contributors to the increases in T2DM prevalence over the study period include ageing populations, increased diagnostic testing for diabetes and an increasing prevalence of obesity^12–14^. The general reductions in T2DM mortality trends observed in this analysis are also likely contributory to the increasing prevalence trends.

These findings suggest that an ongoing public health target should be at primary prevention of T2DM. UK primary care-based screening studies have demonstrated that screening for T2DM identifies 9% of adults with impaired glucose regulation, which represents a state with an increased risk of T2DM and increased mortality^15,16^. Screening for diabetes is also cost effective^17^. Furthermore, evidence from Finland, the UK, the Netherlands and the US has demonstrated the feasibility and efficacy of T2DM prevention programmes^18–20^. The Finnish Diabetes Prevention Study was a randomised controlled trial with four-year follow-up conducted in Finland, which demonstrated a 58% reduction in the risk of T2DM in individuals with impaired glucose tolerance at the end of follow-up in the intervention group compared with controls^18^. The intervention consisted of targeted counselling aimed at reducing weight, reducing fat intake and increasing physical activity. Similarly, a randomised controlled trial of 102 participants with impaired glucose tolerance in the UK, demonstrated an overall reduction in the incidence of T2DM of 55% in the intervention group (receiving targeted motivational interviewing aimed at weight reduction, increased physical activity and reduction of fat intake) when compared with controls over the 3-year study period^21^. Both of the aforementioned randomised controlled trials concluded that T2DM can be prevented in at risk individuals by changes in lifestyle. Despite this, there appears to have been a time lag in the widespread translation of this evidence into clinical practice across the UK and the EU. In 2014, the NHS Diabetes Prevention Programme (NHS DPP) was announced^22^. The programme targets adults in England at risk of T2DM (i.e. with non-diabetic hyperglycaemia), offering evidence-based behavioural interventions (focussed specifically on weight loss, diet and physical activity) in a face-to-face group setting over a minimum of 9 months’ duration. The NHS DPP was rolled out nationally in June 2016 and early outcomes have demonstrated favourable results with widespread engagement. By December 2018, 324,699 referrals to the programme had been made with 152,294 individuals attending the initial assessment^23^. Of the 17,252 individuals who had completed the assessment by December 2018, a mean weight loss of 2.3 kg (95% confidence intervals 2.2–2.3 kg) and mean HbA1C reduction of 1.26 mmol/mol (95% confidence intervals 1.20–1.31 mmol/mol) was identified^23^. The joinpoint analysis for T2DM prevalence performed and presented in this study demonstrates plateauing T2DM prevalence rates since 2015/16–2019 in the UK, with estimated annual percentage increases down to insignificant + 0.9% for males and 0% for females.

The high overall relative increases in prevalence observed in the UK since 1990 may in part be explained by the UK having the largest T2DM mortality reductions of all EU countries for males. There are numerous examples of how a death would be directly attributed to diabetes and registered as such within the GBD Study database: an example provided by the GBD collaborators is a death resulting from acute renal failure, which was caused by a hyperosmolar hyperglycaemic state in an individual with T2DM^24^. The reductions in mortality observed in the UK within the present study were consistent with previous evidence derived from alternative data sources, including The Health Improvement Network (THIN) and the Clinical Practice Research Datalink (CPRD)^25–27^. Being cautious not to infer causality, we suggest that likely contributors to the observed reductions in mortality rates include the improved medical management of the disease once diagnosed, as well as earlier diagnosis and a resultant higher prevalence of early disease. The joinpoint regression analysis performed in the present analysis demonstrates recent plateaus in mortality rates from T2DM in males and females in the UK between 2011 and 2019: explanations for these trends are lacking and should be a focus of future research.

Reducing trends in T2DM mortality were also observed in the majority of the other EU countries between 1990 and 2019, however, trends were less uniform than those observed for prevalence. Indeed, ASMRs increased over the 30-year period studied in 11/28 countries for males and in 4/28 countries for females. Chen et al.^28^ analysed trends in all-cause mortality amongst patients with diabetes (both type 1 and type 2) using data obtained from 35 observational studies identified through systematic review methodology, and identified reductions in all cause mortality rates in the majority of Europid populations with diabetes from 2000 to 2016. The authors also identified that the magnitude of annual mortality reduction matched or exceeded that observed in people without diabetes in nearly 60% of populations studied which reflects well upon the evolving management of individuals diagnosed with diabetes. The data presented in Chen et al.’s systematic review study differs from that derived from the GBD study database in that they do not differentiate between type 1 and type 2 diabetes; they do not isolate mortality resulting directly from diabetes (and instead included all-cause mortality in individuals with diabetes), and they do not include data later than 2016. Despite these methodological differences, both studies identify similar trends towards the majority of Europid populations having reductions in overall mortality over recent decades.

To our knowledge, this is the first paper to compare T2DM prevalence, mortality and DALYs rates across EU countries using data obtained from the 2019 GBD Study. There are, however, several limitations which must be considered when appraising the results of this observational study. Firstly, there may be some inconsistencies both within and between countries when a cause of death is assigned. This is particularly true when considering chronic health conditions like T2DM, as accuracy of cause of death may be confounded by other co-morbidities. However, the quality of health data reporting from the EU countries and the UK is assessed and graded by the GBD Study as discussed in the methodology section of the manuscript, and corrections are made for under-registration. Additionally the GBD uses garbage code redistribution algorithms to improve comparability and reliability (‘garbage’ codes allude to deaths attributed to poorly-defined diagnoses or conditions that cannot be the single underlying cause of death)^3,4^. Secondly, the data obtained is applicable solely for identification and comparison purposes of T2DM across EU countries and, as such, causal relationships cannot be drawn. Third, we accept that changes in coding practices (notably from ICD-9 to ICD-10), as well as changes in the WHO definition of T2DM which occurred during the observation period, may compromise the robustness of the presented data^29^. The GBD maps deaths to causes lists as an attempt to correct for changes in coding systems^30^. Finally, we acknowledge that confounding variables beyond the scope of discussion will have differential effects by country on the data presented from this observational study: using sex-specific, age-standardised mortality, prevalence and DALY rates attempts to account for some of the inevitably confounding variables.

## Conclusion

In summary, there have been general increases in T2DM DALYs and prevalence across EU countries between 1990 and 2019, which have been accompanied by trends towards a reduction in T2DM mortality. Public health measures should continue to focus on primary prevention of T2DM, given its significant costs, morbidity and mortality to affected populations.

## Supplementary Information

Supplementary Tables.

## Data Availability

All data used in this study are freely available online at: http://ghdx.healthdata.org/gbd-results-tool.
