# Supplementary material for: Trends in type 2 diabetes mellitus disease burden in European Union countries between 1990 and 2019
Source: Sci Rep. 2021 Jul 28;11:15356. doi: 10.1038/s41598-021-94807-z (PMC8319179; doi:10.1038/s41598-021-94807-z)
Supplement: Supplementary file 1 — Supplementary Tables. [file 41598_2021_94807_MOESM1_ESM.docx]

**SUPPLEMENTARY MATERIAL**

**Trends in type 2 diabetes disease burden in European Union countries between 1990 and 2019.**

*Richard Goodall^1,2^ 0000-0002-6990-7087, Andrew Alazawi^2,3^ 0000-0001-6564-5828, Will Hughes^2,4^ 0000-0003-0849-671X, Vassiliki Bravis^5^* [*0000-0003-4493-1993*](https://orcid.org/0000-0003-4493-1993)*, Justin D Salciccioli^2,6^ 0000-0001-8092-0883, Dominic C Marshall^2,7^, Conor Crowley^2,8^ 0000-0002-0728-4785, Joseph Shalhoub^2,9^ 0000-0003-1011-7440.*

**Table 1. 1990, 2019 and percentage changes (%) for age-standardised mortality rates (ASMRs), age-standardised prevalence rates (ASPRs) and disability adjusted life years (DALYs) for type 2 diabetes mellitus (T2DM) in all EU countries and the UK for males (95% confidence intervals in brackets).**

| **Country** | **ASMRs** | | | **ASPRs** | | | **DALYs** | | | |
| --- | --- | --- | --- | --- | --- | --- | --- | --- | --- | --- |
|  | **1990** | **2019** | **Change (%)** | **1990** | **2019** | **Change (%)** | **1990** | **2019** | **Change (%)** |  |
| **Austria** | 12.27  (11.5-12.9) | 13.56  (12.3-14.7) | 10.5% | 2195.6 (1923-2481) | 4246.3 (3643-4816) | 93.4% | 402.1  (340-479) | 566.6  (443-721) | 40.9% |  |
| **Belgium** | 9.41  (8.8-10.0) | 6.82  (6.1-7.5) | -27.5% | 2709.4 (2359-3079) | 4504.5 (3940-5068) | 66.3% | 391.7  (309-494) | 496.7 (362-667) | 26.8% |  |
| **Bulgaria** | 14.70  (13.5-15.9) | 15.07  (12.0-18.7) | 2.5% | 3627.6 (3259-4053) | 5494.3 (4848-6095) | 51.5% | 650.2  (533-790) | 802.1  (615-1015) | 23.3% |  |
| **Croatia** | 11.74  (10.8-12.8) | 12.78  (10.2-15.8) | 8.8% | 4212.4  (3761–4680) | 6479.3 (5733-7252) | 53.8% | 608.3  (478-766) | 800.0  (596-1046) | 31.5% |  |
| **Cyprus** | 50.58  (40.6-61.7) | 28.10  (24.1-32.7) | -44.4% | 5445.1 (4744-6126) | 6384.2 (5660-7097) | 17.2% | 1200.4  (994-1438) | 886.4  (709-1097) | -26.2% |  |
| **Czech Republic** | 13.10  (12.0-14.0)) | 16.96  (13.7-20.3) | 29.5% | 5527.7 (4966-6193) | 10198.2 (9038-11335) | 84.5% | 750.7  (576-952) | 1190.5  (879-1556) | 58.6% |  |
| **Denmark** | 11.23  (10.5-12.2) | 14.05  (12.5-15.5) | 25.1% | 2011.2 (1769-2278) | 3826.2 (3413-4231) | 90.2% | 359.9  (313-416) | 493.1  (408-597) | 37.0% |  |
| **Estonia** | 3.62  (3.3-4.0) | 5.00  (3.9-6.3) | 38.1% | 2671.1 (2373-2986) | 4062.7 (3635-4536) | 52.1% | 309.6  (231-400) | 461.5  (337-610) | 49.0% |  |
| **Finland** | 5.96  (5.5-6.2) | 4.18  (3.7-4.6) | -29.9% | 3661.3 (3348-4024) | 6067.8 (5339-6776) | 65.7% | 387.7  (299-495) | 521.9  (371-695) | 34.6% |  |
| **France** | 8.56  (7.9-9.2) | 9.10  (8.2-10.0) | 6.2% | 1781.3 (1600-1965) | 2714.4 (2372-3068) | 52.4% | 261.0  (220-310) | 332.3  (269-409) | 27.3% |  |
| **Germany** | 14.35  (13.5-15.2) | 11.01  (10.0-12.0) | -23.3% | 4467.6 (3922-5024) | 7042.3 (6255-7786) | 57.6% | 568.6  (462-695) | 675.9  (515-868) | 18.9% |  |
| **Greece** | 6.54  (6.11-6.92) | 5.61  (5.1-6.1) | -14.2% | 2787.7 (2483-3133) | 5072.1 (4438-5750) | 81.9% | 349.7  (268-451) | 527.3  (380-701) | 50.8% |  |
| **Hungary** | 10.30  (9.6-10.9) | 13.78  (11.2-16.6) | 33.7% | 4201.3 (3724-4697) | 6889.7 (6120-7658) | 64.0% | 593.9  (466-754) | 885.6  (674-1141) | 49.1% |  |
| **Ireland** | 11.24  (10.4-11.9) | 7.78  (6.8-8.7) | -30.8% | 1589.5 (1383-1804) | 4639.0 (4073-5285) | 191.9% | 311.7  (269-363) | 453.8  (332-602) | 45.6% |  |
| **Italy** | 17.32  (16.6-17.9) | 13.75  (12.4-14.6) | -20.6% | 3930.8 (3476-4368) | 5967.3 (5269-6663) | 51.8% | 582.4  (495-683) | 670.1  (525-837) | 15.1% |  |
| **Latvia** | 4.28  (3.9-4.7) | 7.81  (5.7-9.3) | 82.5% | 2469.7 (2198-2754) | 3991.7 (3563-4443) | 61.6% | 311.5  (237-397) | 520.9  (397-670) | 67.2% |  |
| **Lithuania** | 3.70  (3.4-4.0) | 4.46  (3.6-5.4) | 20.5% | 2443.8 (2162-2734) | 3386.7 (3002-3797) | 38.6% | 293.5  (221-379) | 393.6  (295-523)) | 34.1% |  |
| **Luxembourg** | 10.13  (9.2-11.1) | 6.55  (5.6-7.6) | -35.3% | 1897.0 (1660-2132) | 7001.0 (6239-7825) | 269.1% | 314.3  (263-377) | 626.5  (447-845) | 99.3% |  |
| **Malta** | 21.06  (19.1-23.1) | 13.78  (12.1-15.6) | -34.6% | 4281.4 (3794-4740) | 5757.2 (5122-6451) | 34.5% | 672.9  (559-811) | 658.1  (512-832) | -2.2% |  |
| **Netherlands** | 15.95  (14.9-16.9) | 9.41  (8.4-10.3) | -41.0% | 2823.4 (2497-3144) | 3824.4 (3304-4276) | 35.5% | 526.4  (444-627) | 464.0  (357-594) | -11.9% |  |
| **Poland** | 11.09  (10.4-11.7) | 12.17  (9.8-15.1) | 9.6% | 4243.4 (3843-4658) | 6558.9 (5900-7219) | 54.6% | 634.7  (516-771) | 823.7  (622-1044) | 29.8% |  |
| **Portugal** | 19.57  (18.5-20.5) | 15.92  (14.4-17.3) | -18.7% | 4107.4 (3592-4605) | 6537.9 (3843-4658) | 59.2% | 669.4  (562-799) | 737.9  (573-929) | 10.2% |  |
| **Romania** | 6.61  (6.2-7.0) | 6.85  (5.6-8.1) | 3.6% | 2901.9 (2564-3245) | 4463.9 (3928-5047) | 53.8% | 412.0  (322-523) | 545.4  (409-712) | 32.4% |  |
| **Slovakia** | 12.97  (11.7-14.5) | 8.92  (6.7-11.3) | -31.2% | 3567.9 (3169-3977) | 5010.2 (4436-5589) | 40.4% | 596.3  (478-733) | 622.0  (462-813) | 4.3% |  |
| **Slovenia** | 9.71  (7.6-12.2) | 9.00  (7.1-11.4) | -7.3% | 3774.6 (3345-4192) | 5112.4 (4565-5669) | 35.4% | 518.3  (397-662) | 607.6  (438-797) | 17.2% |  |
| **Spain** | 15.00  (14.1-15.9) | 8.73  (7.8-9.6) | -41.8% | 4229.4 (3916-4584) | 6088.6 (4314-5424) | 44.0% | 599.3  (481-745) | 620.8  (450-832) | 3.6% |  |
| **Sweden** | 9.76 (9.0-10.5) | 9.76  (8.8-10.6) | -0.0% | 2970.6 (2582-3387) | 4670.4 (4090-5241) | 57.2% | 377.4  (307-460) | 477.3  (368-600) | 26.5% |  |
| **United Kingdom** | 9.32  (8.8-9.6) | 4.95  (4.5-5.2) | -46.9% | 3989.2 (3578-4413) | 9120.5 (8177-10209) | 128.6% | 425.9  (342-526) | 683.4  (488-918) | 60.4% |  |

**Table 2. 1990, 2019 and percentage changes (%) for age-standardised mortality rates (ASMRs), age-standardised prevalence rates (ASPRs) and disability adjusted life years (DALYs) for type 2 diabetes mellitus (T2DM) in all EU countries and the UK for females (95% confidence intervals in brackets).**

| **Country** | **ASMRs** | | | **ASPRs** | | | **DALYs** | | | |
| --- | --- | --- | --- | --- | --- | --- | --- | --- | --- | --- |
|  | **1990** | **2019** | **Change (%)** | **1990** | **2019** | **Change (%)** | **1990** | **2019** | **Change (%)** |  |
| **Austria** | 11.74  (10.7-12.5) | 8.83  (7.5-9.9) | -24.8% | 1956.8 (1748-2189) | 3461.5 (3043-3929) | 76.9% | 354.5  (296-430) | 415.7  (316-544) | 19.2% |  |
| **Belgium** | 11.69  (10.5-12.6) | 5.33  (4.6-6.0) | -54.4% | 2484.8 (2184-2796) | 3912.1 (3448-4412 | 57.4% | 395.6  (318-492) | 409.3  (291-550) | 0.1% |  |
| **Bulgaria** | 15.40  (13.8-16.6) | 12.68  (10.2-15.8) | -17.7% | 3203.8 (2866-3567) | 4509.6 (4051-5051) | 40.8% | 625.0  (513-751) | 664.4  (504-839) | -2.7% |  |
| **Croatia** | 11.72  (10.6-12.8) | 9.99  (7.9-12.2) | -14.7% | 3396.1 (3031-3787) | 4911.9 (4355-5596) | 44.6% | 527.6  (420-662) | 608.1  (449-811) | 9.1% |  |
| **Cyprus** | 77.14  (48.5-92.1) | 24.98  (17.5-29.5) | -67.6% | 4525.0 (4059-5028) | 5295.8 (4704-5941) | 17.0% | 1361.7 (1007-1611) | 703.7  (543-883) | -31.2% |  |
| **Czech Republic** | 11.27  (10.1-12.1) | 12.26  (9.9-14.7) | 8.8% | 4171.9 (3697-4628) | 7270.2 (6443-8107) | 74.3% | 598.9  (465-766) | 859.5  (623-1129) | 15.9% |  |
| **Denmark** | 7.71  (7.1-8.4) | 8.15  (7.1-9.2) | 5.6% | 1748.5 (1528-1963) | 3461.2 (3047-3900) | 98.0% | 254.3  (215-300) | 347.8  (272-436) | 31.8% |  |
| **Estonia** | 3.12  (2.7-3.6) | 4.05  (3.1-5.2) | 29.5% | 2339.6 (2074-2605) | 3457.7 (3067-3860) | 47.8% | 276.3  (205-362) | 389.8  (277-520) | 22.4% |  |
| **Finland** | 6.40  (5.7-7.2) | 2.61  (2.2-3.0) | -59.1% | 3387.4 (3036-3745) | 5709.4 (5058-6386) | 68.5% | 356.3  (271-460) | 458.0  (315-626) | 14.8% |  |
| **France** | 7.14  (6.3-7.8) | 5.62  (4.6-6.4) | -21.3% | 1375.0 (1240-1528) | 2283.3 (1988-2621) | 66.1% | 195.9  (164-232) | 231.7  (177-293) | 32.3% |  |
| **Germany** | 15.51  (14.1-16.6) | 8.30  (7.1-9.3) | -46.5% | 4262.6 (3776-4820) | 6232.2 (5520-6924) | 46.2% | 537.4  (437-661) | 532.1  (392-708) | -5.9% |  |
| **Greece** | 8.18  (7.4-8.7) | 4.44  (3.9-4.9) | -45.7% | 2381.6 (2122-2634) | 3870.8 (3361-4351) | 62.5% | 332.9  (261-418) | 392.3  (280-533) | 15.2% |  |
| **Hungary** | 12.22  (11.3-12.9) | 10.02  (8.3-12.2) | -18.0% | 3437.7 (3038-3854) | 4862.6 (4343-5388) | 41.4% | 562.2  (449-688) | 628.4  (473-805) | -4.5% |  |
| **Ireland** | 8.87  (8.1-9.6) | 4.81  (4.1-5.5) | -45.8% | 1340.8 (1162-1538) | 3562.2 (3105-4035) | 165.7% | 244.2  (206-289) | 330.1  (241-438) | 15.6% |  |
| **Italy** | 19.74 (18.2-20.7) | 10.34  (8.6-11.3) | -47.6% | 3509.1 (3106-3898) | 4691.0 (4078-5292) | 33.7% | 575.2  (492-673) | 511.3  (391-651) | -6.1% |  |
| **Latvia** | 4.74  (4.4-5.2) | 7.34  (5.7-9.3) | 55.1% | 2294.3 (2039-2560) | 3558.0 (3189-3939) | 55.1% | 312.3  (240-400) | 464.4  (342-606) | 34.3% |  |
| **Lithuania** | 3.37  (3.1-3.8) | 3.20  (2.6-3.9) | -5.0% | 2059.8 (1846-2307) | 2588.1 (2304-2878) | 25.6% | 256.9  (193-336) | 298.4  (219-397) | 13.2% |  |
| **Luxembourg** | 9.97  (8.8-10.9) | 5.14  (4.2-6.1) | -48.4% | 1744.9 (1542-1960) | 5570.1 (4957-6250) | 219.2% | 284.2  (237-339) | 488.8  (345-662) | 17.8% |  |
| **Malta** | 24.45  (22.0-26.7) | 10.18  (8.4-11.9) | -58.4% | 4149.2 (3712-4650) | 6062.4 (5389-6814) | 46.1% | 706.0  (595-843) | 603.4  (453-787) | -6.0% |  |
| **Netherlands** | 16.55  (14.7-17.9) | 7.56  (6.4-8.5) | -54.3% | 2351.9 (2084-2623) | 2962.4 (2359-3079) | 26.0% | 458.6  (387-537) | 340.3  (256-436) | -7.5% |  |
| **Poland** | 12.51  (11.6-13.3) | 8.83  (7.0-10.8) | -29.4% | 3820.8 (3457-4194) | 4867.2 (4316-5403) | 27.4% | 623.8  (508-756) | 603.0  (452-778) | -3.1% |  |
| **Portugal** | 19.98  (18..5-21.2) | 13.27  (5.9-8.2) | -33.6% | 3871.1 (3416-4338) | 6100.6 (5336-6891) | 57.6% | 638.8  (540-760) | 638.5  (481-823) | 6.2% |  |
| **Romania** | 6.40  (6.0-6.8) | 5.20  (4.2-6.3) | -18.8% | 2365.7 (2090-2673) | 3423.4 (2991-3841) | 44.7% | 365.9  (289-456) | 426.5  (318-558) | 10.7% |  |
| **Slovakia** | 12.15  (10.8-13.6) | 7.48  (5.8-9.4) | -38.5% | 2913.4 (2598-3248)) | 4001.3 (3575-4461) | 37.3% | 506.9  (409-622) | 505.2  (376-666) | -2.5% |  |
| **Slovenia** | 10.64  (8.3-13.2) | 5.61  (4.2-7.2) | -47.2% | 3238.8 (2901-3607) | 3934.4 (3504-4417) | 21.5% | 476.2  (366-608) | 449.4  (322-596) | -0.4% |  |
| **Spain** | 18.30  (16.2-19.7) | 7.26  (5.9-8.2) | -60.3% | 3782.1 (3389-4141) | 4837.6 (4314-5424) | 27.9% | 602.0  (486-737) | 490.6  (347-666) | -19.7% |  |
| **Sweden** | 7.36  (6.5-8.1) | 6.34  (5.4-7.1) | -13.9% | 2440.8 (2132-2772) | 3732.4 (3234-4234) | 52.9% | 285.3  (232-349) | 341.7  (259-444) | 20.5% |  |
| **United Kingdom** | 7.06  (6.4-7.4) | 3.73  (3.3-4.0) | -47.1% | 3171.7 (2834-3524) | 6806.3 (6093-7582) | 114.6% | 325.6  (259-407) | 499.2  (355-667) | 35.9% |  |
